# The invasion of abandoned fields by a major alien tree filters understory plant traits in novel forest ecosystems

**DOI:** 10.1038/s41598-018-26493-3

**Published:** 2018-05-30

**Authors:** T. Sitzia, T. Campagnaro, D. J. Kotze, S. Nardi, A. Ertani

**Affiliations:** 1 Department of Land, Environment, Agriculture and Forestry, Università degli Studi di Padova, Viale dell’Università 16, I-35020 Legnaro, PD Italy; 2 Faculty of Biological and Environmental Sciences, Ecosystems and Environment Research Programme, University of Helsinki, Niemenkatu 73, FIN-15140 Lahti, Finland; 3 Department of Agronomy, Food, Natural resources, Animals and Environment, Università degli Studi di Padova, Viale dell’Università 16, I-35020 Legnaro, PD Italy

## Abstract

The abandonment of agricultural use is a common driver of spontaneous reforestation by alien trees. The N-fixing black locust (*Robinia pseudoacacia* L.) is a major alien invader of old fields in Europe. Here we show that canopy dominance by this tree may filter the frequency distribution of plant functional traits in the understory of secondary woodlands. Higher soil C/N ratio and available P are associated with black locust stands, while higher soil phenols associate with native tree stands. These environmental effects result in differences in understory flowering periods, reproduction types and life forms. Our findings emphasize the effect of a major alien tree on functional plant trait composition in the early stages of spontaneous reforestation of abandoned lands, implying the development of a novel forest ecosystem on a large geographical scale.

## Introduction

A reduction in anthropogenic pressure is commonly regarded as beneficial to biodiversity^1^. However, the cessation of a certain land use might either lead to a recovery or a loss of biodiversity in response to changes in physical and biological processes. For example, spontaneous reforestation that follows the interruption of agricultural practices has been common throughout contemporary Europe as a result of socio-economic drivers^2^. While this has brought about wilderness recovery, most of the traditional heterogeneous landscape and valuable open habitats, like grasslands, meadows and extensive pastures, have been lost^3^ or have frequently undergone transformation into novel ecosystems composed of a mixture of native and alien species^4,5^.

Relative to native ecosystems, alien plant invasion generally produces higher above-ground net primary production and litter decomposition, and an increase in ecosystem carbon (C) and nitrogen (N) pools due to positive feedbacks between invasion and biogeochemical cycles^6,7^. Alien plant invasions across large spatial scales, such as those produced by the widespread colonisation of abandoned agricultural lands by alien trees in Europe, might influence ecosystem processes and determine habitat and resources for other taxa at these large scales. This is particularly true for N-fixing trees, which show greater retention of older soil C in afforested agricultural lands than in neighbouring woodlands without these trees^8^. Such impacts on nutrient cycling are likely to affect plant species and communities^9^, and composition in functional traits^10^.

Identifying which plant traits in the understory strongly vary between alien and native tree stands can elucidate how the invasive trees affect functional community composition. Using a functional rather than a species-level perspective can also generalize results to species that share common trait syndromes^11^. A functional trait approach helps to assess how the dominance of alien trees might affect cultivation legacy, either increasing or decreasing the duration of the effects of past agricultural practices. Changes in plant trait composition could either limit or enhance the ability of ecosystems to resist or recover from disturbances or to provide beneficial ecosystem goods and services^12^. In turn, the legacy of cultivation will determine the likelihood that management actions on old invaded fields will either produce a historical or natural vegetation state, or a degraded state that will be resistant to restoration^13^.

The nitrogen-fixing (N-fixing) alien black locust (*Robinia pseudoacacia* L.), hereafter also referred to as “alien”, is among the most common and widespread successful colonisers of abandoned sites^14,15^. After establishment, black locust increases soil organic matter, is able to modify the availability of nutrients, particularly N, and has a variable effect on total phosphorous (P), available P and potassium in the topsoil^16,17^. Its canopy architecture might change the partitioning of rainfall among interception loss, infiltration and runoff. Therefore, this species is the perfect candidate to study the effects of an alien invader on biodiversity at continental scales. The effects of black locust on plant communities have been demonstrated by focusing on individual species. Compared to sites colonised by native trees, those colonised by black locust differ in plant^18,19^, bird^20^, lichen^21^ and soil biotic^17^ communities. Differences vary with stand age, landscape composition and management^22^. Little is known about black locust’s effects on the frequency distribution of functional traits in these communities^23^.

**Figure 1:**
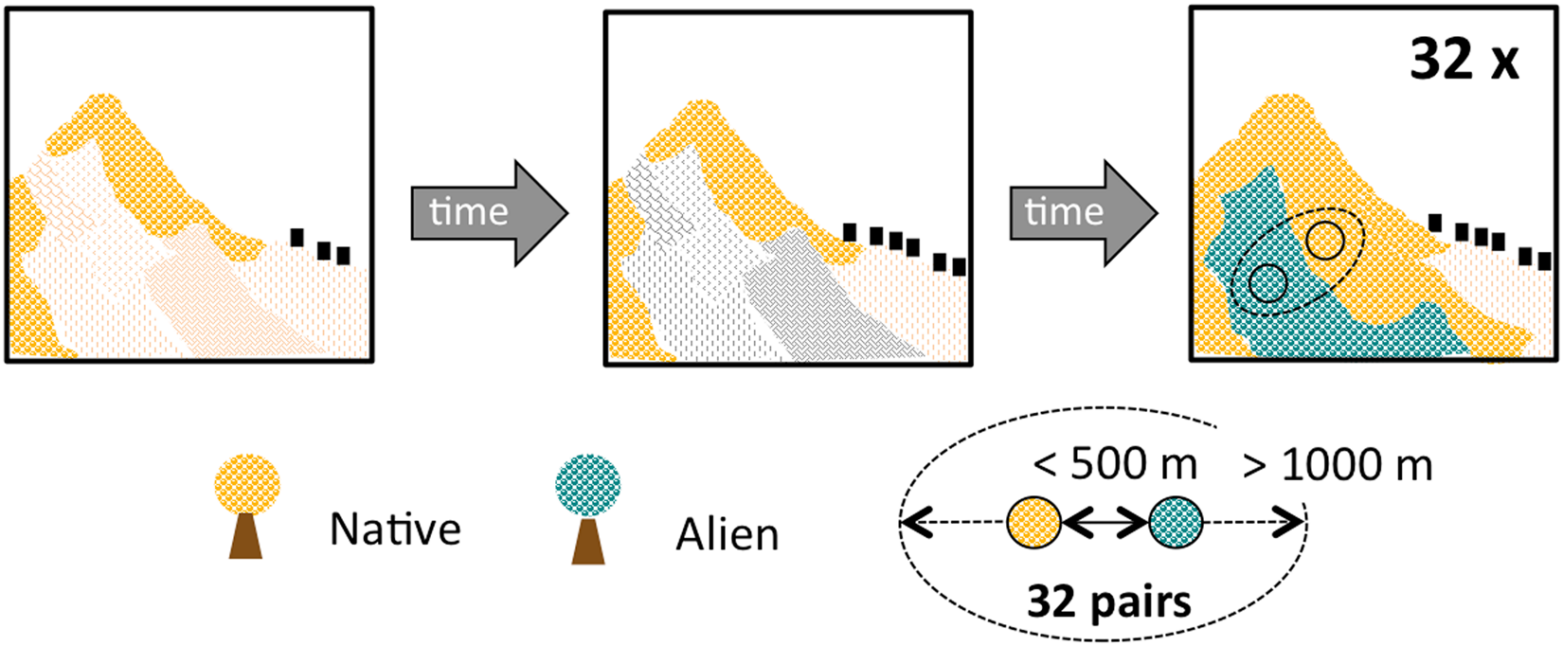
Sampling design. Schematic representation of the paired sampling design: 32 pairs of black locust vs. native stands were selected. Each of the 64 stands developed on former agricultural land (orange colour) during the last 35–40 years, which have been abandoned (grey colour) due to marginality or conversion to urban land uses (black rectangles). Members of each pair were less than 500 m apart, and more than 1 km apart from other pairs.

Here we investigate the understory vegetation of neighbouring black locust and native tree woodlands, in pairs, across a vast northern Mediterranean lowland region under agricultural land-use abandonment. We ascertained that both stands (black locust and native) of each pair belonged to the same former land-use and that this has been abandoned in the previous 35–40 years (Fig. 1). This sampling design ensures that the stand pairs developed under similar site and land cover conditions and avoids spatial autocorrelation effects^24^. We use RLQ analysis to assess co-correlations between topographical, soil, stand, and land cover variables (R table) and species trait attributes (Q table), constrained by the relative abundances of understory vegetation species (L table). We also perform a partial RLQ to separate the effects of canopy dominance (native vs. alien) on understory trait distributions from those representing environmental conditions^25,26^.

The dominance of N-fixing black locust in the canopy should result in a higher accumulation of soil C, N and available P compared to adjacent stands where the canopy is dominated by native trees. Plant-soil interactions should have a major control on the functional trait composition of understory vegetation, selecting against slow growing species with short dispersal in favour of fast growing plants. Even though abiotic environmental filters (e.g. land cover or site conditions) could influence functional trait composition, we expect that black locust dominance in the canopy, per se, should produce measurable variation in plant trait composition in the understory compared to woodlands dominated by native trees. Should the set of measurable traits in black locust woodlands be altered from the original neighbouring woodlands, they could be considered novel ecosystems^27^.

## Results

Global testing showed statistical significance for both the basic and partial RLQ, indicating a global relationship between environmental variables and plant traits. The percentage of total co-inertia explained by the first two axes of the basic and the partial RLQ (i.e. respectively including or not including canopy dominance) was 66% and 69%, respectively. The first axis of the basic RLQ explained an almost equal proportion of co-inertia than this axis in the partial RLQ (51% vs. 53%) (Supplementary Table S1).

**Figure 2:**
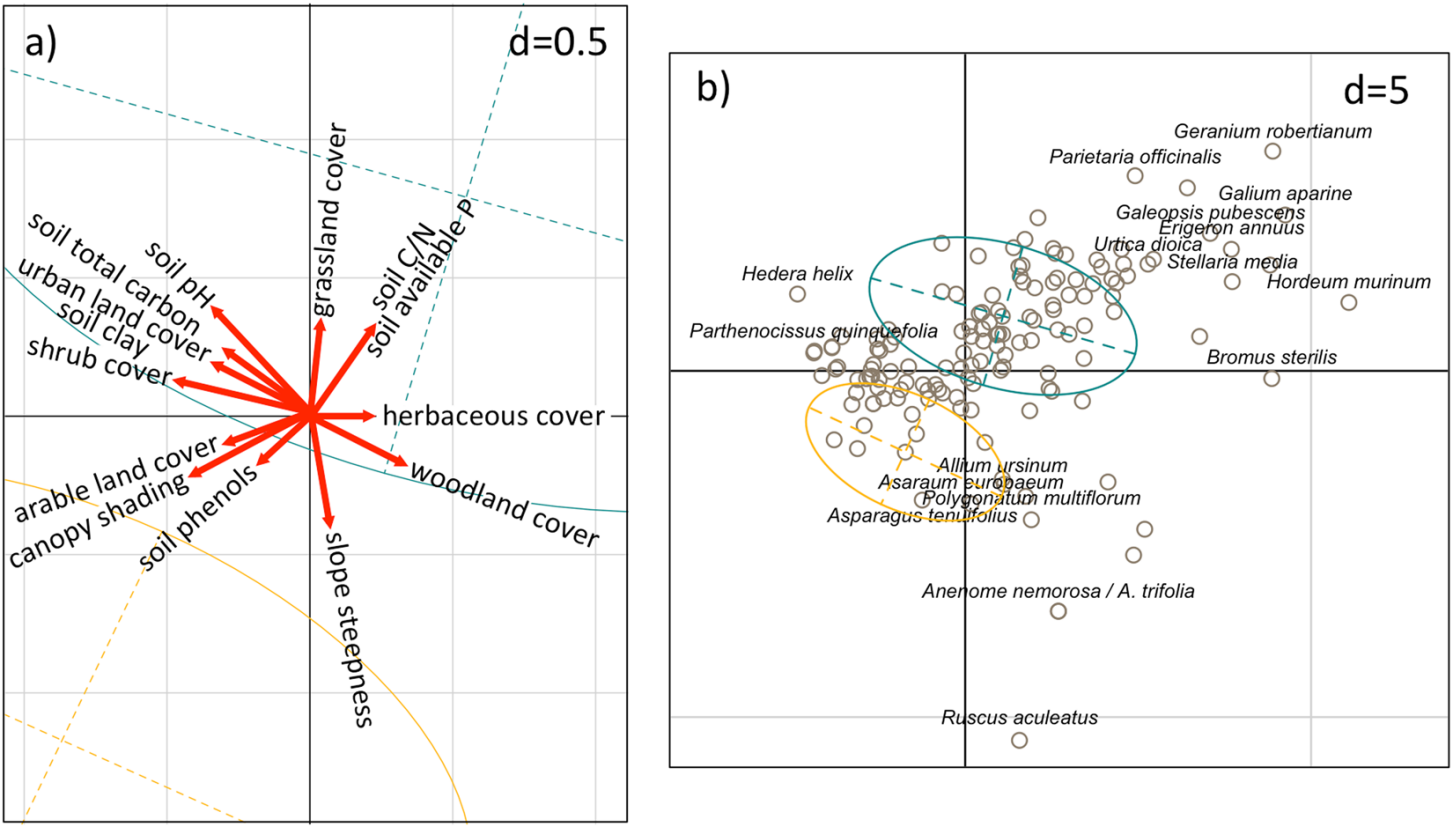
RLQ ordination diagrams of environmental variables and species. Plant species responses to environmental gradients and canopy dominance by alien vs. native trees. Ordination diagrams of the first two axes of the RLQ-analysis displaying (**a**) scores of the most significant environmental variables and (**b**) species scores. d represents grid size. Ellipses represent dispersion of the alien (cyan) and native (golden) tree dominance in the stand canopy (see Fig. 4).

**Figure 3:**
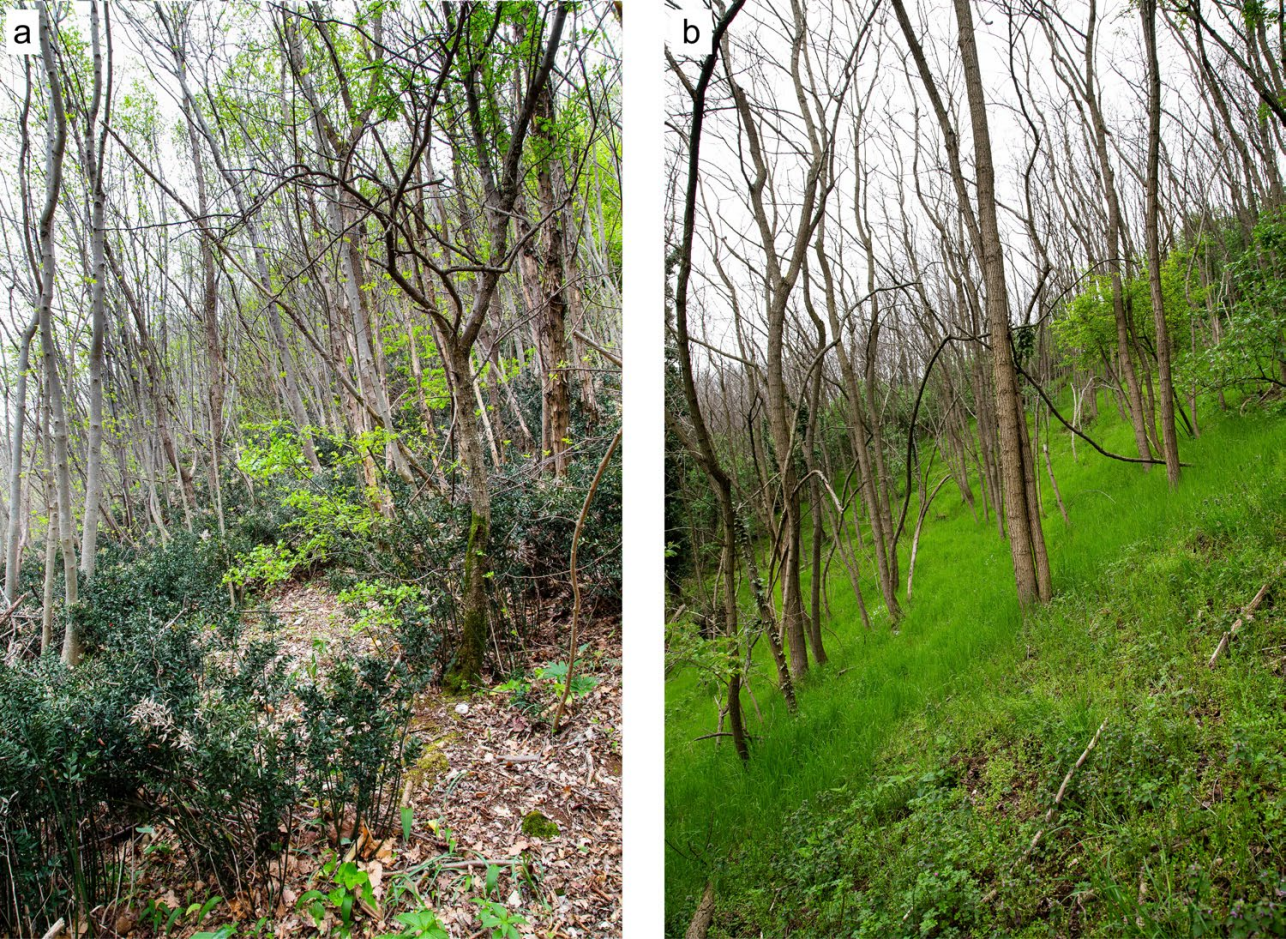
A comparative figure of understories under canopies dominated by native vs. black locust trees. An example is presented of how the canopy of secondary woodlands dominated by native trees such as *Fraxinus ornus* (**a**) might develop very different understories from those found under canopies dominated by the alien black locust tree (**b**) (Photos by G. Corradini and T. Sitzia).

Several variables were correlated with one or both of the basic RLQ axes (Supplementary Table S2). The first basic RLQ axis was positively correlated with herbaceous cover, and negatively with shrub cover and canopy shading (lower light availability). Among the land cover types, woodlands were correlated positively with the first basic RLQ axis, while urban and cropland cover negatively, indicating an edge disturbance intensity gradient along this axis. Grassland cover was positively correlated with the second basic RLQ axis. A soil condition gradient was represented by a decrease in soil clay, total C and pH along the first axis, and an increase in the soil C/N ratio, available P and a reduction in phenols (soil N mineralization) and slope steepness along the second axis (Fig. 2a and Supplementary Table S2). The soil condition gradient along the second axis was also reflected in a vegetation gradient, from shade-tolerant forest species, like *Anemone nemorosa*, *A. trifolia*, *Allium ursinum*, *Asarum europaeum*, *Asparagus tenuifolius*, *Polygonatum multiflorum*, *Ruscus aculeatus* and *Vinca minor* to competitive species indicative of disturbance, soil fertility and weedy habitats, like *Erigeron annuus*, *Galeopsis pubescens*, *Galium aparine*, *Geranium robertianum*, *Parietaria officinalis*, *Stellaria media*, *Urtica dioica* and annual or biennial grasses, like *Hordeum murinum* and *Bromus sterilis* (Figs 2b and 3). Moreover, an increase of shrub cover, particularly by *Hedera helix* and *Parthenocissus quinquefolia*, which formed extensive carpets, reduced the cover of herbaceous species, thought this was not strictly correlated with a decrease in light availability for the understory (Fig. 2b).

**Figure 4:**
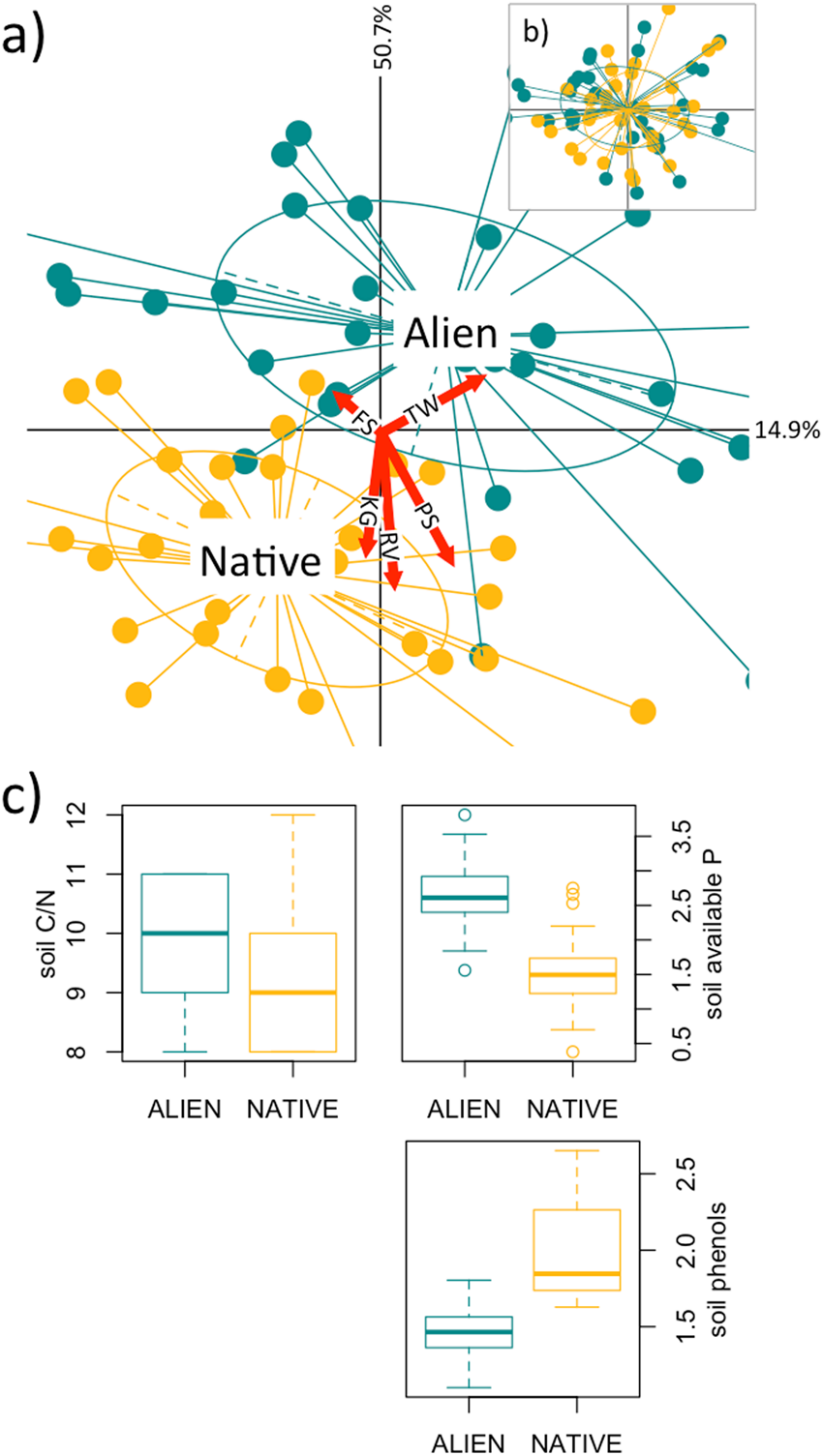
Trait responses to alien vs. native tree canopy. Trait responses to alien vs. native tree dominance in the stand canopy (TW = overwintering, PS = self-pollination, RV = vegetative reproduction, KG = geophytes, FS = flowering during January-March). Sample scores (64 stands) of the first two axes of the basic RLQ (**a**) and partial RLQ (**b**). Samples are marked after the alien (cyan) vs. native (golden) stratification. The size and direction of the most significant plant trait effects are represented by red arrows. Boxplots (**c**) represent the distribution of soil properties mostly related to the alien vs. native stands (median, inner-quartile range (IQR), minimum and maximum values < 1.5 IQR and outliers > 1.5 × IQR).

The ordination diagram of the basic RLQ showed the overwhelming influence of canopy dominance, since nearly all of the black locust stands clustered at the top half of the plot (Fig. 4a). In the partial RLQ, where the effect of canopy dominance was removed, this clustering disappeared (Fig. 4b), overall significance was maintained, but either the significance, or the contribution to total inertia of some variables were lost, namely the C/N ratio and available P in the soil, but also phenols which had a negligible contribution to total inertia in the partial RLQ (Fig. 4c and Supplementary Table S2).

**Figure 5:**
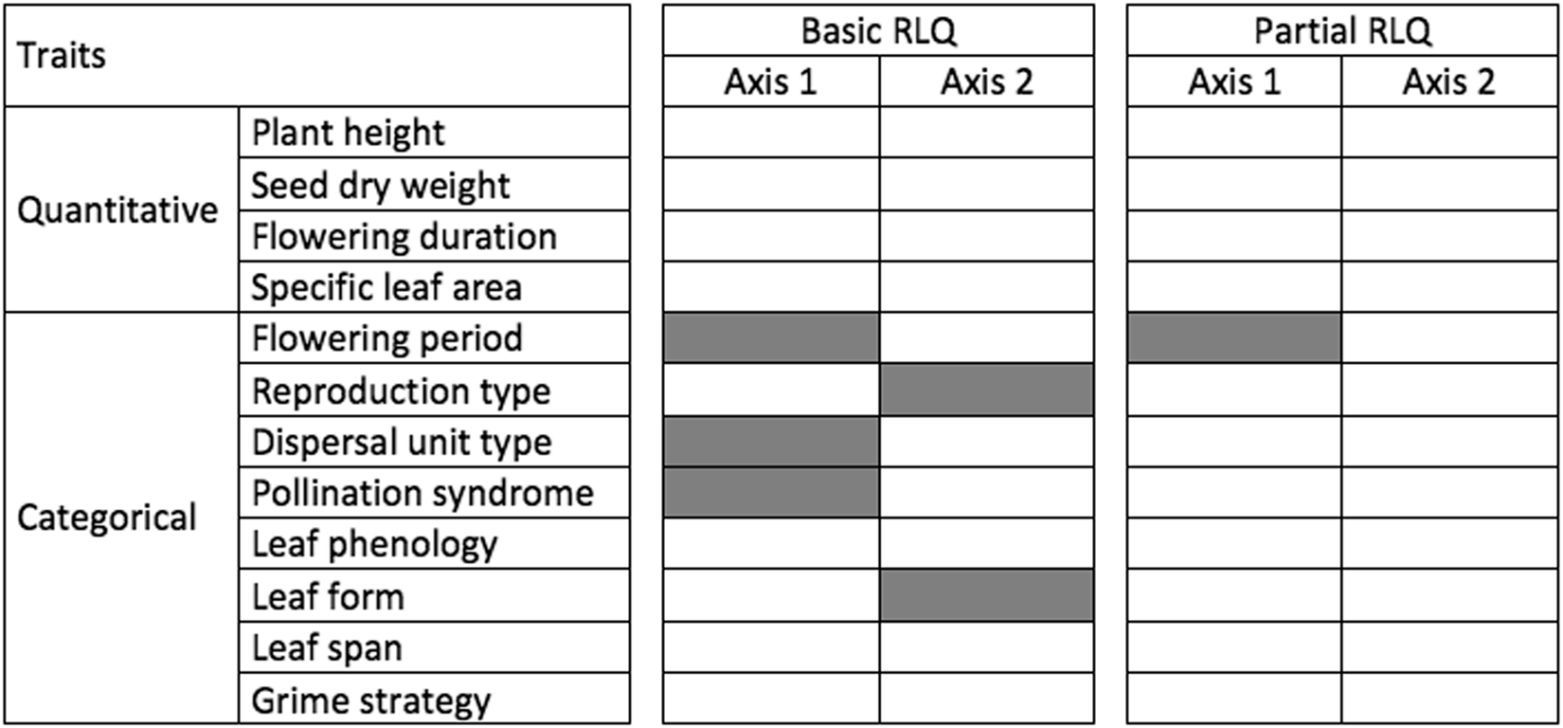
Relationships between traits and RLQ axes. Schematic representation of the relationship between traits and the first two axes of the basic (left) and partial (right) RLQ resulting from the fourth-corner analysis. Grey cells indicate significant associations of traits and axes. The significance of the association between a trait and one axis of the partial RLQ was combined from two-fourth-corner objects and compared at P = 0.05.

Among the environmental variables, the roles of herbaceous cover, urban and grassland cover and slope steepness became insignificant in the partial RLQ. Moreover, fewer plant traits were significantly correlated with the partial RLQ axes, and only flowering period was still significantly associated with one of these axes (Fig. 5 and Supplementary Table S3). In particular, the plant traits that best represented the contrast between alien vs. native canopy were late flowering season and overwintering, associated with alien stands, while vegetative reproduction, self-pollination, and geophytes were associated with native stands (Fig. 4a).

This indicates that a considerable amount of variability expressed by plant traits is related to processes resulting from the dominance of either black locust or native trees in the canopy.

## Discussion

### General patterns

Our study shows that plant trait variability in the understory of alien vs. native tree canopies is mainly driven by three processes: soil N mineralization, light availability and edge disturbance intensity. The process of soil N mineralization must mostly be driven by the dominance of black locust in the canopy, given that this tree species contributes to an increase in the C/N ratio and available P and a decrease in phenols in the soil. Light availability and edge disturbance intensity were also associated, to some extent, to the canopy dominance by black locust vs. native trees, but less strictly, as is shown by the partial RLQ results.

### Soil processes

Plant invasion can stimulate the fixation of a large amount of atmospheric CO_2_ into the ecosystem^6^. N-fixing trees, such as black locust, typically accumulate more C in soils than forests without N-fixing trees^8^. The differential effects within a range of tree species potentially encroaching former cultivated fields produce corresponding variation to the time length of the legacy induced by former agricultural practices^28^. In fact, the observed difference between alien and native stands may develop from fundamentally different processes, with either greater accumulation of recently fixed C or reduced decomposition of older soil C^8,29^.

Contrasting results have been reported about the ability of black locust to increase organic C and N, as well as P, in the soil. Some authors have shown an increase in these soil properties^16^, which contribute to higher leaf quality and growth rate^30^. Others have shown that black locust does not alter rates of potential N mineralization, nor shifts the amount of soil mineral N^31^. Microbial activity might be inhibited by black locust litter due to secondary compounds^32^. Rice *et al*.^33^, however, pointed out elevated N-mineralization rates in nutrient poor ecosystems, associated with low-lignin leaf litter. Also, De Marco *et al*.^34^ showed that black locust plantations have a higher ratio of water soluble organic matter than paired conifer plantations, increasing C sequestration in the mineral soil.

In our study area, former land cover types were grasslands or arable lands, which change soil fertility differently. Grasslands are represented by slightly moist meadow or arid communities, which have been either manured or grazed, or both, in the past. They are expected to be more fertile than arable lands, therefore their soil organic carbon (SOC) must be higher^35^. On the contrary, a considerable amount of SOC is expected to be lost by crop harvesting and erosion in arable fields during cultivation^36^. In former arable fields, C and total N content in soil and the C/N ratio have been found to be lower than in semi-natural habitats, both at local^37^ and global levels^38^. The same contrasts have been found with organic P, whose loss is much higher with cropping than in adjacent permanent pastures^39^. This is particularly true with cereals, which are the most common arable crop in the study area, because their low P-concentrated residues may even reduce P availability due to assimilation in the microbial biomass^40^. On the contrary, perennial hay meadows develop labile organic P pools available to plants, in place of occluded inorganic P^41^.

Depending on fertility of the former agricultural soil, also in our study area, black locust should accumulate greater amounts of recently-fixed C than native stands or reduces decomposition of older soil C^8,29^. This is confirmed by higher values of the soil C/N ratio observed in black locust stands, supporting what has been found in black locust stands compared with native oak stands in China^36^ and in aging black locust plantations^42^. Moreover, black locust either reduces the losses of available P of former manured semi-natural grasslands or increase its availability in former arable lands. In other words, the association of black locust to available P and the soil C/N ratio might be either direct or indirect, black locust could be either a passenger or a driver of the environmental changes which follows land use abandonment^23^.

Much higher values of available P have been observed in Central European black locust woodlands, except for steep and grassy sites^14^. In the same sites, higher N values, but similar values for the soil C/N ratio, have been measured. The studied Central European black locust stands have been established for at least 40 years, in some cases for over a century^14^. Ours are relatively younger, their soils are still impoverished by the recent agricultural past, which is a possible explanation for the observed differences. However, like in Central Europe^14^, organic matter decomposition rate should be fast in black locust stands, faster than in the corresponding woodlands dominated by native trees.

### Plant trait patterns

Our results agree with Graae and Sunde^43^ who compared ancient vs. recent forests and with Lososová *et al*.^44^ who found that in sites more frequently colonised by black locust stands, overwintering green species are more common. Terwei *et al*.^23^ also found that woodlands dominated by black locust may slow down forest succession by establishing new communities with a dense grass-dominated understory.

Moreover, our results suggest a filter on species that flower during mid-summer, mediated by the rapid decomposition of black locust leaves and P-abundant flowers during the summer^45^, while early flowering has been related to a short and unpredictable growing season^46^. This is also related to the fact that the position of black locust stands in the basic RLQ ordination was opposite to canopy shading. This confirms that native trees might develop more shading crowns than black locust^20,47^. Shade stress is avoided by early flowering species, because they complete the above-ground stage of their life cycles before the forest canopy closes and have more time to develop seeds. On the contrary, late-growing species in less shading black locust stands have an advantage in light interception and growth temperature, with high reproductive capacity^48^, also thanks to higher availability of soil P and a higher soil C/N ratio.

Hipps *et al*.^49^ observed that both ancient shade-tolerant forest species, like *A. nemorosa*, and shade-tolerant competitive species, like *U. dioica*, seem to benefit or tolerate high soil P concentrations, but the former suffers from competition by the latter, hindering the establishment of semi-natural woodland vegetation on fertile and former agricultural soils that have residual concentrations of P. We observed something similar in our study area, a positive feedback between soil N mineralization, P availability and plant traits associated with higher reproductive capacity. We also showed an association of phenols with canopy dominance by native trees, which in turn affect their plant trait composition and confirms their role in litter decomposition, N mineralization, and plant-soil interactions^50^ and their indication of more natural conditions^51^.

**Figure 6:**
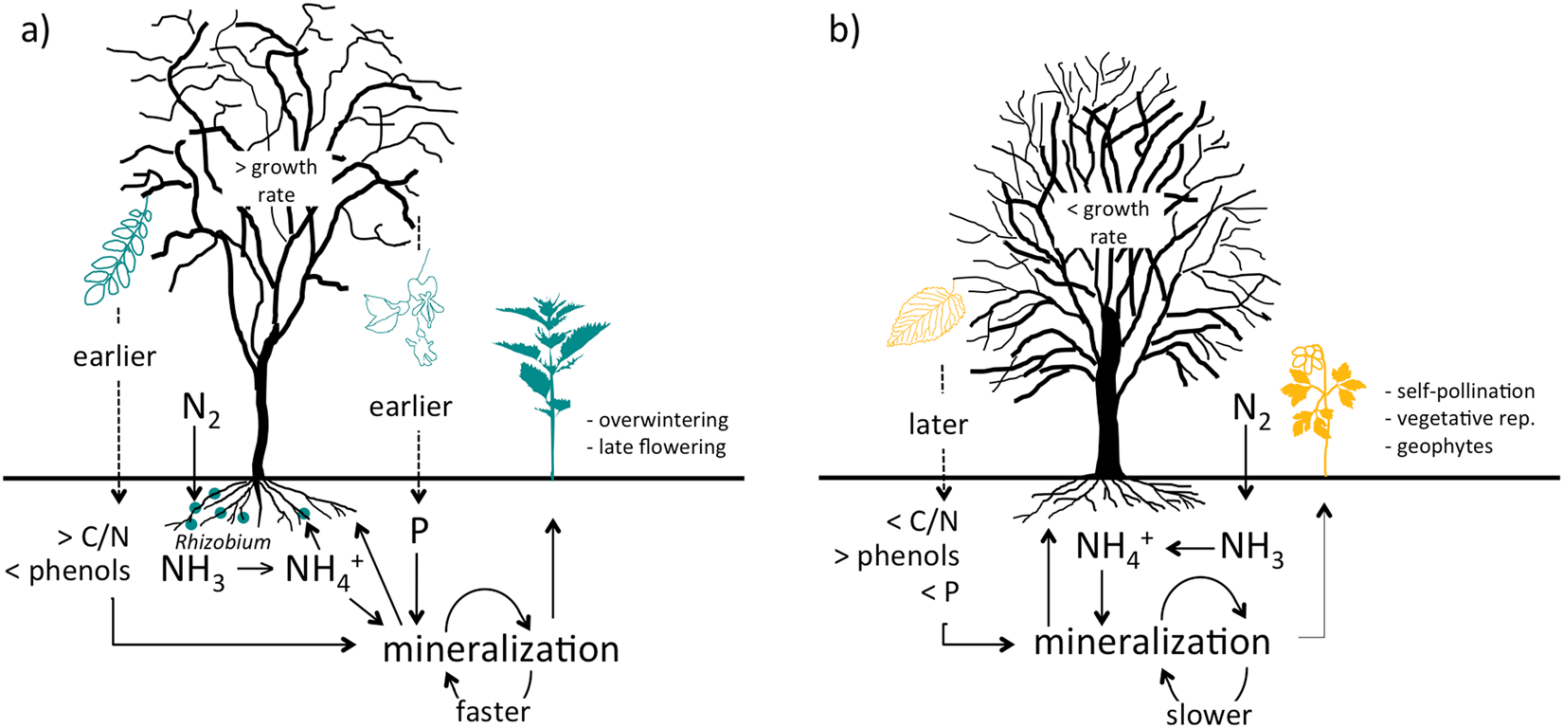
Graphical abstract. A conceptual representation showing the observed relationship between black locust (**a**) and native tree (**b**) canopy dominance, environmental variables and plant trait composition.

In fact, species that reproduce vegetatively or are self- and insect-pollinated, and with early and short flowering times, like geophytes, were associated with the native tree canopy, as well as with lower values of soil available P and C/N ratio, and higher values of soil phenols. Species with mid-summer flowering and overwintering were associated with the alien tree canopy, and with opposite values of the three soil properties (Fig. 6). This does not imply that, during some phenological phases, black locust stands could not display specific understory plants, such as geophytes^47^, but, observed during the same time period than native stands, the latter would be richer in geophytes.

## Conclusions

Previous studies have shown that understory species α diversity does not vary between recent black locust and native stands on former rural agricultural lands, and only β diversity of specific understory plant guilds differ slightly, while tree diversity is strongly affected^24^. Here we show that a higher soil C/N ratio and available P are associated with black locust and in turn to differences in flowering period, reproduction type and life form of understory plants. Black locust has the ability to either conserve or increase available P and organic C stocks in soils, which, within 35 years of colonisation, favour mid-summer flowering and overwintering green plants. Recent secondary alien black locust stands should be regarded as “novel ecosystems”, emphasizing their effects on plant functional trait composition rather than species diversity in the early stages of spontaneous reforestation of abandoned land. Implication at the landscape scale is a generalised shift to mid-summer flowering, with reduced early and late flowering plants and vegetative reproduction, and self- and insect pollination, especially related to geophytes.

We caution against the increased availability of organic P, which may increase the loss of P in runoff, effectively turning black locust P sinks into P sources^52^. Moreover, afforestation in general, produces an increase in soil C stocks, 30–50 years after afforestation, therefore we expect further increases in C and N stocks in alien stands in the future^53^.

Further research is needed to assess (i) seasonal variability of the effect of black locust on light availability to the understory, ii) the direct rather than indirect association of black locust to soil available P, the soil C/N ratio, and related plant trait composition, iii) the persistence in time and space of the observed patterns, and iv) consequent management implications. Moreover, it will be crucial to combine the analysis of dry and fresh soil samples and to directly measure mineralization rates with experimental designs, which also include litter.

## Material and Methods

### Study area and sampling design

Data were collected from a vast northern Mediterranean study area located in the south-eastern pre-Alps and neighbouring lowlands of the Padana Plain (mean altitude: 250 m a.s.l.). Climate ranges from humid subtropical on the Padana Plain to oceanic in the pre-Alps. Mean annual rainfall and temperature are 1000 mm and 12 °C, respectively^54^. The study area encompasses approximately 5000 km^2^.

Within the study area, we selected 32 pairs of black locust vs. native forest stands, having a mean tree canopy cover of 66% (30–95%). Each of the 64 stands developed on former agricultural lands during the last 35–40 years (Fig. 1). The basal area of black locust trees (as a proportion) was used to distinguish between the two types of stands, respectively 0–15% (mean = 2%) in native stands and 42–100% (mean = 87%) in black locust stands. The tree layer of the native stands is characterised by the following species^24^ (n = number of stands): *Fraxinus ornus* (n = 5), *Quercus pubescens* and *Q. petraea* (n = 10), *Ostrya carpinifolia* (n = 6), *Alnus glutinosa*, *Castanea sativa* and *Acer pseudoplatanus* (n = 6) and *Ulmus minor* (n = 5).

Members of each pair were less than 500 m apart, and more than 1 km apart from other pairs. Pairs were surveyed at the same time, from early May to late July 2011, to avoid bias due to intra-pair decoupling of phenological phases.

### Environmental data

Understory vegetation, topographical, stand, land cover and soil variables were collected from each stand (Supplementary Table S4). We measured vascular plant species cover from one 100 m^2^ plot inside each stand using the cover classes of Braun-Blanquet^55^, which were later approximated to Tüxen & Ellenberg cover percentages^56^. We limited the survey to understory plants lower than 1 m in height. Topographical variables included slope steepness, southness and elevation. Southness was measured as (180° - |aspect - 180°|). The age of the oldest tree in a stand was approximated to the age of the largest tree, among those developed after land-use abandonment, and measured counting the annual tree rings from a core taken at the stump base. Cover percentages of the tree, shrub and herbaceous layers were visually estimated. Canopy shading was measured with a solar power meter as 1 minus the average of four solar irradiance measures at the understory level relative to irradiance outside the edge of the tree shade. Canopy density was measured with a spherical densiometer. Mean diameter at breast height and tree height were measured with a caliper and a hypsometer, from which total and specific basal areas are later calculated. Former land cover was classified as semi-natural (grasslands) and artificial (arable and urban lands) from the interpretation of historical available images of 1978 to 1983.

The covers of woodland, grassland, arable and urban land were measured within 250, 500 and 1000 m from each sampling unit in 1988–89, using historical aerial photographs^57^, and in 2007 using an existing soil use map^58^. On one hand, the cover of woodland and grassland vs. arable and urban land should be interpreted as a contrast between buffering against indirect human disturbance vs. high levels of edge disturbance intensity, respectively^59^. On the other hand, the cover of grassland vs. arable land are associated, respectively, with the colonisation of semi-natural vs. artificial former agricultural uses, and their corresponding former management regimes.

### Soil data analysis

We collected three soil subsamples at approximately 0–20 cm depth, in randomly selected locations at least 2 m apart inside the stands. Vegetation residues, grass and litter, if any, were previously removed. The three subsamples were bulked to create a 500-g sample and put in a labelled plastic bag. Samples were air dried and analysed afterwards. Soil pH was measured potentiometrically using 1:2.5 soil/water extracts. Total C content was determined by the calcimeter method and gravimetric loss of CO_2_. Particle size analysis was performed according to the hydrometer method, using sodium hexametaphosphate as a dispersant^60^. Electrical conductivity was measured with a conductivity sensor, in a suspension of soil and distilled water at a ratio 1:5. Soil organic C and total N were determined using a dry combustion procedure in an element analyser (vario MACRO CNS, Hanau, Germany) and a factor of 1.72 was used to convert organic C to organic matter. Plant available P was calculated using the Olsen method^61^. Humic substances were extracted with 0.1 M NaOH (1:10 w/v); the suspension was shaken for 16 h at room temperature in a N_2_ atmosphere and freed from the suspended material by centrifugation at 7000 g for 20 min. Subsequently, the solution was filtered through a column of Amberlite IR-120 hydrogen form and the extract was dialyzed in Wisking tubes against distilled water to pH 6.0. Water soluble phenols were extracted with distilled water^62,63^. Thirty grams of dry weight samples were mixed in 200 ml distilled water and shaken at 75 rpm for 20 h at room temperature. Solutions were filtered through Whatman’s No. 1 paper. All samples were extracted in triplicate. Total water-soluble phenols (monomeric and polyphenols) were determined by using the Folin-Ciocalteau reagent, following the method of Box^64^. Catechin was used as a standard and the concentration of water-soluble phenolic compounds was expressed as nmol cat/mg.

The measures described above were performed on the three subsamples collected at each stand and were later averaged.

### Plant traits

Classes within categorical traits summed to 36 trait attributes (Supplementary Table S3). For species recorded at least once with a cover class ≥5%, we searched for plant traits in the TRY database^65^ and other sources. Plant height, seed dry weight, onset of the flowering season, flowering duration, specific leaf area, dispersal unit type, life span, reproduction, pollination syndrome, leaf phenology, Raunkier’s life form, and Grime’s competitive strategy were collected at species level. The species-by-trait matrix included 144 plant species with four quantitative traits and eight categorical traits (Supplementary Table S5).

### Statistical analysis

Paired t-test and Wilcoxon signed-rank test were used to evaluate differences in the measured environmental variables between black locust and native stands. Percentage N in the soil, soil organic C and matter correlated strongly with each other (r ≥ 0.9), as expected^66^, so too did land cover types between different buffer zones and periods. Therefore, among these correlated variables, we retained only soil organic matter and the percentages of land cover types of the historical aerial photographs within a 500 m radius around each stand for further analysis. We performed two RLQ analyses similar to Wesuls *et al*.^67^, using the ade4 package for the R statistical environment^68^. A basic RLQ was performed where canopy dominance (representing native vs. black locust stands) was one of the variables. Canopy dominance was also considered an environmental filter and a partial RLQ was performed to separate the effects of canopy dominance (here native vs. alien tree canopy) on understory trait distributions from those representing physical and biological processes related to environmental conditions. In other words, we evaluated the change in importance of plant traits (Q-table) when variation in the R (environmental variables × samples) and L (species × samples) tables that were linked to black locust dominance, was removed^67^.

We were interested in the correlations of environmental variables and traits with the basic RLQ axes, but also with the partial RLQ axes representing vectors controlled for canopy dominance (native vs. alien tree canopy). Therefore, comparing the contribution of each environmental variable to total inertia in the basic and the partial RLQ should reveal the most important environmental gradients in each analysis. Further, the contribution to total inertia could also be used as a measure of relevance of traits when comparing results of the two RLQs. In particular, variables that lose importance in the partial RLQ are related more to processes associated with the dominance of black locust vs. native trees in the canopy, while those that maintain or gain importance are mostly related to other processes.

## Electronic supplementary material

Supplementary information

Supplementary Table S5

## References

[1] Newbold T (2015). Global effects of land use on local terrestrial biodiversity. Nature.

[2] MacDonald D (2000). Agricultural abandonment in mountain areas of Europe: Environmental consequences and policy response. J. Environ. Manage..

[3] Sitzia T, Semenzato P, Trentanovi G (2010). Natural reforestation is changing spatial patterns of rural mountain and hill landscapes: a global overview. Forest Ecol. Manag..

[4] Chytrý M (2009). European map of alien plant invasions based on the quantitative assessment across habitats. Divers. Distributions.

[5] Campagnaro, T., Brundu, G. & Sitzia, T. Five major invasive alien tree species in European Union forest habitat types of the Alpine and Continental biogeographical regions. *J. Nat. Conserv*. 10.1016/j.jnc.2017.07.007 (2017).

[6] Liao CZ (2008). Altered ecosystem carbon and nitrogen cycles by plant invasion: a meta-analysis. New Phytol..

[7] Castro-Díez P, Godoy O, Alonso A, Gallardo A, Saldaña A (2014). What explains variation in the impacts of exotic plant invasions on the nitrogen cycle? A meta-analysis. Ecol. Lett..

[8] Resh SC, Binkley D, Parrotta JA (2002). Greater soil carbon sequestration under nitrogen-fixing trees compared with *Eucalyptus* species. Ecosystems.

[9] Vilà M (2011). Ecological impacts of invasive alien plants: a meta-analysis of their effects on species, communities and ecosystems. Ecol. Lett..

[10] Fischer FM, Oliveira JM, Dresseno ALP, Pillar VD (2014). The role of invasive pine on changes of plant composition and functional traits in a coastal dune ecosystem. Nat. Conservacao.

[11] Vandewalle M (2010). Functional traits as indicators of biodiversity response to land use changes across ecosystems and organisms. Biodivers. Conserv..

[12] Lavorel S (2011). Using plant functional traits to understand the landscape distribution of multiple ecosystem services. J. Ecol..

[13] Cramer VA, Hobbs RJ, Standish RJ (2008). What’s new about old fields? Land abandonment and ecosystem assembly. Trends Ecol. Evol..

[14] Vítková M, Tonika J, Müllerová J (2015). Black locust—Successful invader of a wide range of soil conditions. Sci. Total Environ..

[15] Camenen E, Porté AJ, Benito Garzón M (2016). American trees shift their niches when invading Western Europe: evaluating invasion risks in a changing climate. Ecol. Evol..

[16] Qiu LP, Zhang XC, Cheng JM, Yin XQ (2010). Effects of black locust (*Robinia pseudoacacia*) on soil properties in the loessial gully region of the Loess Plateau, China. Plant Soil.

[17] Lazzaro L (2018). How ecosystems change following invasion by *Robinia pseudoacacia*: Insights from soil chemical properties and soil microbial, nematode, microarthropod and plant communities. Sci. Total Environ..

[18] Trentanovi G (2013). Biotic homogenization at the community scale: disentangling the roles of urbanization and plant invasion. Divers. Distributions.

[19] Benesperi R (2012). Forest plant diversity is threatened by *Robinia pseudoacacia* (black-locust) invasion. Biodivers. Conserv..

[20] Hanzelka J, Reif J (2015). Responses to the black locust (*Robinia pseudoacacia*) invasion differ between habitat specialists and generalists in central European forest birds. J. Ornithol..

[21] Nascimbene J, Nimis PL, Benesperi R (2012). Mature non-native black-locust (*Robinia pseudoacacia* L.) forest does not regain the lichen diversity of the natural forest. Sci. Total Environ..

[22] Sitzia T, Campagnaro T, Kowarik I, Trentanovi G (2016). Using forest management to control invasive alien species: helping implement the new European regulation on invasive alien species. Biol. Invasions.

[23] Terwei A (2016). Response of floodplain understorey species to environmental gradients and tree invasion: a functional trait perspective. Biol. Invasions.

[24] Sitzia T, Campagnaro T, Dainese M, Cierjacks A (2012). Plant species diversity in alien black locust stands: A paired comparison with native stands across a north-Mediterranean range expansion. Forest Ecol. Manag..

[25] Dolédec S, Chessel D, Ter Braak CJF, Champely S (1996). Matching species traits to environmental variables: a new three-table ordination method. Environ. Ecol. Stat..

[26] Gámez-Virués S (2015). Landscape simplification filters species traits and drives biotic homogenization. Nat. Commun..

[27] Hobbs RJ, Higgs E, Harris JA (2009). Novel ecosystems: implications for conservation and restoration. Trends Ecol. Evol..

[28] Cuesta B, Benayas JR, Gallardo A, Villar-Salvador P, González-Espinosa M (2012). Soil chemical properties in abandoned Mediterranean cropland after succession and oak reforestation. Acta Oecol..

[29] Wang F (2010). Effects of nitrogen-fixing and non-nitrogen-fixing tree species on soil properties and nitrogen transformation during forest restoration in southern China. Soil Sci. Plant Nutr..

[30] Moshki A, Lamersdorf NP (2011). Growth and nutrient status of introduced black locust (*Robinia pseudoacacia* L.) afforestation in arid and semi arid areas of Iran. Res. J. Environ. Sci..

[31] Castro-Diez P, Gonzalez-Munoz N, Alonso A, Gallardo A, Poorter L (2009). Effects of exotic invasive trees on nitrogen cycling: a case study in Central Spain. Biol. Invasions.

[32] Castro-Diez P, Fierro-Brunnenmeister N, Gonzalez-Munoz N, Gallardo A (2012). Effects of exotic and native tree leaf litter on soil properties of two contrasting sites in the Iberian Peninsula. Plant Soil.

[33] Rice SK, Westerman B, Federici R (2004). Impacts of the exotic, nitrogen-fixing black locust (*Robinia pseudoacacia*) on nitrogen-cycling in a pine-oak ecosystem. Plant Ecol..

[34] De Marco A, Esposito F, Berg B, Giordano M, De Santo AV (2013). Soil C and N sequestration in organic and mineral layers of two coeval forest stands implanted on pyroclastic material (Mount Vesuvius, South Italy). Geoderma.

[35] Lugato E, Bampa F, Panagos P, Montanarella L, Jones A (2014). Potential carbon sequestration of European arable soils estimated by modelling a comprehensive set of management practices. Glob. Change Biol..

[36] Tateno R (2007). Comparison of litterfall production and leaf litter decomposition between an exotic black locust plantation and an indigenous oak forest near Yan’an on the Loess Plateau, China. Forest Ecol. Manag..

[37] Kopecky M, Vojta J (2009). Land use legacies in post-agricultural forests in the Doupovské Mountains, Czech Republic. Appl. Veg. Sci..

[38] Guo LB, Gifford RM (2002). Soil carbon stocks and land use change: a meta analysis. Glob. Change Biol..

[39] Hedley MJ, Stewart JWB, Chauhan BS (1982). Changes in inorganic and organic soil phosphorus fractions induced by cultivation practices and by laboratory incubations 1. Soil Sci. Soc. Am. J..

[40] Damon PM, Bowden B, Rose T, Rengel Z (2014). Crop residue contributions to phosphorus pools in agricultural soils: A review. Soil Biol. Biochem..

[41] Crews TE, Brookes PC (2014). Changes in soil phosphorus forms through time in perennial versus annual agroecosystems. Agric. Ecosyst. Environ..

[42] Li H (2013). Changes in carbon, nutrients and stoichiometric relations under different soil depths, plant tissues and ages in black locust plantations. Acta Physiol Plant..

[43] Graae BJ, Sunde PB (2000). The impact of forest continuity and management on forest floor vegetation evaluated by species traits. Ecography.

[44] Lososová Z (2006). Patterns of plant traits in annual vegetation of man-made habitats in central Europe. Perspect. Plant Ecol. Evol. Syst..

[45] Lee YC, Nam JM, Kim JG (2011). The influence of black locust (*Robinia pseudoacacia*) flower and leaf fall on soil phosphate. Plant Soil.

[46] Weinig C, Schmitt J (2004). Environmental effects on the expression of quantitative trait loci and implications for phenotypic evolution. BioScience.

[47] Vitkova M, Muellerova J, Sadlo J, Pergl J, Pysek P (2017). Black locust (*Robinia pseudoacacia*) beloved and despised: A story of an invasive tree in Central Europe. Forest Ecol. Manag..

[48] Sun S, Frelich LE (2011). Flowering phenology and height growth pattern are associated with maximum plant height, relative growth rate and stem tissue mass density in herbaceous grassland species. J. Ecol..

[49] Hipps NA, Davies MJ, Dodds P, Buckley GP (2005). The effects of phosphorus nutrition and soil pH on the growth of some ancient woodland indicator plants and their interaction with competitor species. Plant Soil.

[50] Kuiters AT (1990). Role of phenolic substrances from decomposing forest litter in plant-soil interactions. Acta Bot. Neerl..

[51] Sitzia T (2014). Topsoil organic matter properties in contrasted hedgerow vegetation types. Plant Soil.

[52] Dodd RJ, Sharpley AN (2015). Recognizing the role of soil organic phosphorus in soil fertility and water quality. Resour. Conserv. Recycl..

[53] Medina-Villar S (2016). Impacts of the alien trees *Ailanthus altissima* (Mill.) Swingle and *Robinia pseudoacacia* L. on soil nutrients and microbial communities. Soil Biol. Biochem..

[54] ARPAV. *Il clima in Veneto*, http://www.arpa.veneto.it/temi-ambientali/climatologia/approfondimenti/il-clima-in-veneto (2011).

[55] Braun-Blanquet, J. Pflanzensoziologie. Grundzüge der Vegetationskunde. (Springer, 1928).

[56] Maarel van der E (2007). Transformation of cover-abundance values for appropriate numerical treatment – Alternatives to the proposals by Podani. J. Veg. Sci..

[57] Geoportale Nazionale. *Ortofoto in bianco e nero anni 1988-1989 con relative date del volo*, http://wms.pcn.minambiente.it/ogc?map=/ms_ogc/WMS_v1.3/raster/ortofoto_bn_88.map&service=wms&request=getCapabilities&version=1.3.0 (2013).

[58] Regione del Veneto. *L.R. n. 28/76 - Formazione della Carta Tecnica Regionale*, https://www.regione.veneto.it/web/ambiente-e-territorio/condizioni-di-utilizzo-geoportale (2017).

[59] Harper KA (2005). Edge influence on forest structure and composition in fragmented landscapes. Conserv. Biol..

[60] Gee, G. W. & Bauder, J. W. in *Methods of soil analysis: Part 1—Physical and mineralogical methods* Vol. 5.1 *SSSA* Book Series (ed Arnold Klute) 383–411 (Soil Science Society of America, American Society of Agronomy, 1986).

[61] Olsen, S. R. & Dean, L. A. in Metho*ds* of s*oi*l ana*lysis. Part 2. Chemical and microbiological properties* Vol. 9.2 *Agronomy Monograph* (ed A. G. Norman) 1035–1049 (American Society of Agronomy, Soil Science Society of America, 1965).

[62] Kaminsky R, Müller WH (1978). A recommendation against the use of alkaline soil extractions in the study of allelopathy. Plant Soil.

[63] Kaminsky R, Müller WH (1977). The extraction of soil phytotoxins using a neutral EDTA solution. Soil Sci..

[64] Box JD (1983). Investigation of the Folin-Ciocalteau phenol reagent for the determination of polyphenolic substances in natural waters. Water Res..

[65] Kattge J (2011). TRY – a global database of plant traits. Glob. Change Biol..

[66] Yang Y, Luo Y, Finzi AC (2011). Carbon and nitrogen dynamics during forest stand development: a global synthesis. New Phytol..

[67] Wesuls D, Oldeland J, Dray S (2012). Disentangling plant trait responses to livestock grazing from spatio-temporal variation: the partial RLQ approach. J. Veg. Sci..

[68] Dray S, Dufour AB (2007). The ade4 package: implementing the duality diagram for ecologists. J. Stat. Softw..

