# Supplementary material for: The invasion of abandoned fields by a major alien tree filters understory plant traits in novel forest ecosystems
Source: Sci Rep. 2018 May 30;8:8410. doi: 10.1038/s41598-018-26493-3 (PMC5976623; doi:10.1038/s41598-018-26493-3)
Supplement: Supplementary file 1 — Supplementary information [file 41598_2018_26493_MOESM1_ESM.docx]

**The invasion of abandoned fields by a major alien tree filters understory plant traits in novel forest ecosystems**

T. Sitzia^1*^, T. Campagnaro.^1^, D. J. Kotze^2^, S. Nardi.^3^ & A. Ertani^3^

^1^ Department of Land, Environment, Agriculture and Forestry, Università degli Studi di Padova, Viale dell'Università 16, I-35020 Legnaro (PD), Italy

^2^ Faculty of Biological and Environmental Sciences, Ecosystems and Environment Research Programme, University of Helsinki, Niemenkatu 73, FIN-15140 Lahti, Finland

^3^ Department of Agronomy, Food, Natural resources, Animals and Environment, Università degli Studi di Padova, Viale dell'Università 16, I-35020 Legnaro (PD), Italy

* Corresponding author (correspondence to tommaso.sitzia@unipd.it)

**Supplementary information**

Supplementary Table S1. Eigenvalues, percentage and cumulative percentage of variance (Cum %) explained by the first five axes of the basic (sum of eigenvalues: 8.108) and partial (sum of eigenvalues: 6.691) RLQ.

| Axis | Basic RLQ | | | Partial RLQ | | |
| --- | --- | --- | --- | --- | --- | --- |
|  | Eigenvalue | % | Cum % | Eigenvalue | % | Cum % |
| 1 | 4.115 | 50.7 | 50.7 | 3.560 | 53.1 | 53.2 |
| 2 | 1.210 | 14.9 | 65.7 | 1.077 | 16.1 | 69.3 |
| 3 | 0.949 | 11.7 | 77.4 | 0.494 | 7.4 | 76.7 |
| 4 | 0.415 | 5.1 | 82.5 | 0.398 | 5.9 | 82.6 |
| 5 | 0.386 | 4.8 | 87.3 | 0.300 | 4.5 | 87.1 |

Supplementary Table S2. Percentage contribution to total inertia in the basic and partial RLQ, and Pearson correlations (r) of environmental variables with axes 1 and 2 of the basic and partial RLQ according to fourth-corner statistics (* represents p < 0.1 and ** p < 0.05).

| Environmental variable | Contribution to total inertia [%] | | Correlation r with basic RLQ axes | | Correlation r with partial RLQ axes | |
| --- | --- | --- | --- | --- | --- | --- |
|  | Basic RLQ | Partial RLQ | Axis 1 | Axis 2 | Axis 1 | Axis 2 |
| Canopy dominance |  |  |  |  |  |  |
| Alien | 2.14 | - | 0.19* | 0.23* | - | - |
| Native | 2.85 | - | -0.19* | -0.23* | - | - |
| Topographical variables |  |  |  |  |  |  |
| Slope steepness | 2.73 | 3.15 | 0.05 | -0.21* | -0.08 | 0.20 |
| Southness | 1.63 | 1.98 | 0.06 | -0.05 | -0.07 | 0.00 |
| Elevation | 2.24 | 2.66 | 0.06 | 0.10 | -0.06 | -0.17 |
| Stand variables |  |  |  |  |  |  |
| Age of the largest tree | 6.54 | 7.03 | -0.26* | -0.21 | 0.24* | 0.25** |
| Tree layer cover | 0.93 | 0.95 | -0.06 | -0.08 | 0.05 | 0.10 |
| Shrub layer cover | 8.69 | 9.72 | -0.40** | 0.06 | -0.38** | -0.09 |
| Herbaceous layer cover | 2.51 | 1.89 | 0.19** | 0.00 | -0.17 | 0.06 |
| Tree canopy shading | 7.53 | 7.95 | -0.35** | -0.12 | 0.34** | 0.18 |
| Tree canopy density | 5.20 | 6.17 | -0.26* | -0.00 | 0.26* | 0.12 |
| Mean tree DBH | 2.69 | 2.14 | 0.01 | 0.12 | 0.03 | 0.03 |
| Mean tree height | 1.17 | 1.13 | 0.05 | 0.04 | -0.04 | -0.00 |
| Tree basal area | 1.93 | 2.59 | -0.09 | -0.02 | 0.10 | 0.12 |
| Land cover variables |  |  |  |  |  |  |
| Semi-natural former land use | 1.93 | 2.29 | 0.08 | 0.11 | -0.06 | -0.04 |
| Woodland cover | 4.69 | 5.60 | 0.28** | -0.09 | -0.28** | 0.01 |
| Grassland cover | 2.49 | 3.04 | 0.02 | 0.17* | -0.01 | -0.19 |
| Cropland cover | 2.52 | 2.99 | -0.17* | -0.05 | 0.18 | 0.12 |
| Urban cover | 3.71 | 4.60 | -0.22** | 0.08 | 0.24 | -0.02 |
| Soil variables |  |  |  |  |  |  |
| pH (KCl) | 6.15 | 7.56 | -0.28* | 0.20 | 0.31* | -0.11 |
| Conductivity | 0.51 | 0.65 | 0.00 | -0.00 | -0.01 | -0.02 |
| Total carbon | 4.35 | 5.55 | -0.26* | 0.11 | 0.27* | -0.06 |
| Sand | 0.97 | 1.12 | 0.06 | 0.05 | -0.04 | -0.00 |
| Silt | 4.18 | 5.08 | 0.24* | -0.14 | -0.25* | 0.11 |
| Clay | 4.89 | 4.90 | -0.29** | 0.10 | 0.29** | -0.10 |
| Organic matter | 1.57 | 1.72 | -0.00 | -0.02 | -0.02 | -0.08 |
| C/N | 3.14 | 1.53 | 0.17* | 0.17* | -0.13 | -0.12 |
| Humic carbon | 4.09 | 4.94 | 0.08 | -0.20* | -0.10 | 0.21 |
| Available P | 3.27 | 0.43 | 0.16* | 0.14 | -0.11 | -0.01 |
| Phenols | 2.57 | 0.67 | -0.15 | -0.09 | -0.11 | -0.02 |

Supplementary Table S3. Percentage contribution to total inertia of the basic and partial RLQ (each column sums up to 100%) and Pearson correlations of quantitative and categorical traits with axes 1 and 2 of the basic and partial RLQ according to fourth-corner statistics (combination of models 2 and 4, after Dray and Legendre 2008).

| Plant trait / attribute | | Contribution to total inertia [%] | | Correlation r with basic RLQ axis | | Correlation r with partial RLQ axes | |
| --- | --- | --- | --- | --- | --- | --- | --- |
|  |  | Basic RLQ | Partial RLQ | Axis 1 | Axis 2 | Axis 1 | Axis 2 |
| Quantitative traits | |  |  |  |  |  |  |
|  | Plant height | 4.09 | 4.30 | -0.27 | 0.07 | 0.25* | -0.08 |
|  | Seed dry weight | 1.62 | 1.36 | -0.09 | -0.00 | 0.05 | -0.10 |
|  | Flowering duration | 1.95 | 1.24 | 0.02 | 0.18 | 0.05 | -0.03 |
|  | Specific leaf area | 3.40 | 2.50 | 0.22 | 0.15 | -0.16 | -0.09 |
|  | |  |  |  |  |  |  |
| Categorical traits | |  |  |  |  |  |  |
| Onset flowering season | |  |  |  |  |  |  |
|  | January-March | 1.99 | 2.12 | 0.02 | -0.24 | -0.07 | 0.22 |
|  | April-May | 1.85 | 1.29 | -0.19 | -0.04 | 0.13 | -0.06 |
|  | June-July | 8.05 | 7.66 | 0.45** | 0.10 | -0.41** | -0.16 |
|  | August-September | 3.85 | 4.75 | -0.27** | 0.13 | 0.33* | 0.05 |
| Reproduction type | |  |  |  |  |  |  |
|  | Mostly by seed | 1.43 | 1.66 | 0.04 | -0.05 | -0.07 | -0.04 |
|  | Mostly vegetative | 3.34 | 3.25 | 0.04 | -0.36** | -0.12 | 0.31 |
|  | By seed or spore | 0.77 | 0.68 | -0.07 | 0.03 | 0.04 | -0.10 |
|  | By seed and vegetative | 1.29 | 0.66 | 0.01 | 0.23 | 0.07 | -0.08 |
| Dispersal unit type | |  |  |  |  |  |  |
|  | Fruit or infructescence | 2.83 | 3.20 | -0.28 | -0.10 | 0.28 | 0.20 |
|  | Fruitlet | 0.93 | 1.13 | 0.08 | -0.02 | -0.09 | -0.04 |
|  | Mericarp or part of infructescence | 0.86 | 0.95 | 0.04 | 0.09 | -0.02 | -0.08 |
|  | Aggregate fruit | 3.16 | 3.46 | 0.20 | -0.04 | -0.19 | 0.00 |
|  | Seed | 1.69 | 2.06 | -0.10 | 0.04 | 0.11 | 0.04 |
|  | Spore or vegetative | 0.37 | 0.43 | 0.07 | 0.00 | -0.07 | -0.03 |
|  | Fruit with appendage | 4.11 | 4.55 | 0.28 | 0.08 | -0.26 | -0.14 |
|  | Part of fruit | 1.70 | 1.51 | -0.13 | 0.01 | 0.09 | -0.10 |
| Pollination syndrome | |  |  |  |  |  |  |
|  | Insect | 4.34 | 4.97 | -0.39** | 0.11 | 0.40** | -0.03 |
|  | Selfing | 4.65 | 5.50 | 0.21** | -0.28 | -0.22 | 0.34 |
|  | Wind | 2.42 | 2.94 | 0.14 | 0.05 | -0.16 | -0.16 |
|  | Insect and wind or insect and selfing and wind or selfing and wind | 2.39 | 2.90 | 0.12 | 0.03 | -0.13 | -0.14 |
|  | Selfing and insect | 1.28 | 1.36 | 0.14 | 0.01 | -0.12 | -0.02 |
| Leaf phenology | |  |  |  |  |  |  |
|  | Summer green | 1.35 | 1.13 | 0.01 | -0.02 | -0.07 | -0.20* |
|  | Spring green | 3.11 | 3.59 | 0.03 | -0.05 | 0.00 | 0.14 |
|  | Persistent green | 1.38 | 1.67 | -0.15 | -0.01 | 0.18 | 0.17 |
|  | Overwintering green | 4.37 | 3.19 | 0.27* | 0.10 | -0.21 | 0.00 |
| Life form | |  |  |  |  |  |  |
|  | Chamaephyte | 2.06 | 2.07 | -0.07 | -0.02 | 0.03 | 0.00 |
|  | Geophyte | 2.75 | 2.41 | -0.01 | -0.33** | -0.07 | 0.27* |
|  | Hemicryptophyte | 2.35 | 1.80 | 0.18 | 0.16 | -0.13 | -0.14 |
|  | Phanerophyte | 1.64 | 1.83 | -0.25 | -0.07 | 0.24 | -0.09 |
|  | Therophyte | 3.60 | 2.67 | 0.25 | 0.10 | -0.19 | 0.00 |
| Life span | |  |  |  |  |  |  |
|  | Perennial | 0.29 | 0.21 | -0.24 | -0.10 | 0.18 | 0.00 |
|  | Annual or biennial | 3.54 | 2.59 | 0.24 | 0.10 | -0.19 | 0.00 |
| Grime competitive strategy | |  |  |  |  |  |  |
|  | R and CR | 3.81 | 4.38 | 0.23 | -0.19 | -0.23 | 0.26 |
|  | C | 1.45 | 1.32 | 0.02 | -0.05 | -0.08 | -0.18 |
|  | CS | 3.24 | 3.98 | -0.25 | 0.16 | 0.30 | 0.00 |
|  | CRS | 0.68 | 0.73 | 0.06 | 0.05 | -0.04 | -0.02 |

Supplementary Table S4.

Topographical, stand, land cover and soil variables used in the R-table: groups, symbols, descriptions and units; mean/or frequency and standard error (SE) of each variable distinguished between native, alien and all stands. For quantitative variables, the p-value of a paired t-test for normally distributed data (in bold) and of a Wilcoxon paired test for non-normally distributed data (in plain text) are also reported (Test). P-values < 0.10 are presented in italics.

| **Groups** | **Symbol** | **Description and unit** | **Native stands** | | **Alien stands** | | **All stands** | | **Test** |
| --- | --- | --- | --- | --- | --- | --- | --- | --- | --- |
|  |  |  | **Mean/ Freq.** | **SE** | **Mean/ Freq.** | **SE** | **Mean/ Freq.** | **SE** |  |
| Topographic variables | SLO | Slope steepness (°) | 15.13 | 1.39 | 14.03 | 1.77 | 14.59 | 1.12 | **0.487** |
|  | SOU | Southness (180° - \|aspect - 180°\|) | 159.61 | 15.30 | 165.23 | 17.43 | 162.42 | 11.51 | **0.804** |
|  | ELE | Elevation (m a.s.l.) | 251.22 | 25.75 | 244.35 | 28.49 | 247.79 | 19.05 | ***0.079*** |
| Stand variables | TAG | Age of the largest tree (y) | 22.94 | 0.84 | 21.78 | 1.13 | 22.36 | 0.70 | **0.467** |
|  | TRC | Tree layer cover (%) | 69.69 | 2.87 | 61.88 | 2.79 | 65.78 | 2.05 | *0.089* |
|  | SHC | Shrub layer cover (%) | 54.38 | 3.45 | 50.78 | 4.57 | 52.58 | 2.85 | 0.493 |
|  | HEC | Herbaceous layer cover (%) | 59.38 | 4.63 | 79.38 | 2.79 | 69.38 | 2.96 | *0.004* |
|  | TSH | Tree canopy shading (0-1) | 0.86 | 0.01 | 0.83 | 0.02 | 0.85 | 0.01 | 0.339 |
|  | TCC | Tree canopy cover (%) | 89.92 | 0.90 | 89.64 | 0.96 | 89.78 | 0.65 | 0.844 |
|  | DBH | Mean tree DBH (cm) | 10.11 | 0.34 | 12.25 | 0.55 | 11.18 | 0.35 | ***0.001*** |
|  | THE | Mean tree height (m) | 9.67 | 0.41 | 10.79 | 0.45 | 10.23 | 0.31 | ***0.040*** |
|  | TBA | Tree basal area (m^2^ ha^-1^) | 20.03 | 1.59 | 24.53 | 2.18 | 22.28 | 1.37 | **0.145** |
| Land cover variables | NAT | Semi-natural former land-use (0/1) | 19 |  | 19 |  | 38 |  |  |
|  | HWX | Proportion of land cover types H: historical (1981-83) R: recent (2007) W: woodland G: grassland A: cropland U: urban 250 (2) -500 (5) -1000 (X)  m buffer radius | 35.56 | 3.06 | 35.01 | 3.14 | 35.29 | 2.17 | **0.519** |
|  | HGX |  | 15.05 | 1.70 | 14.67 | 1.72 | 14.86 | 1.20 | 0.105 |
|  | HAX |  | 33.82 | 3.23 | 34.01 | 3.24 | 33.92 | 2.27 | **0.794** |
|  | HUX |  | 14.85 | 1.40 | 15.46 | 1.51 | 15.16 | 1.02 | **0.346** |
|  | HW5 |  | 39.59 | 2.91 | 40.04 | 3.38 | 39.82 | 2.21 | **0.786** |
|  | HG5 |  | 18.95 | 2.26 | 18.01 | 2.34 | 18.48 | 1.62 | 0.153 |
|  | HA5 |  | 29.08 | 3.48 | 29.13 | 3.39 | 29.10 | 2.41 | **0.925** |
|  | HU5 |  | 11.94 | 1.19 | 12.35 | 1.57 | 12.14 | 0.98 | **0.650** |
|  | HW2 |  | 46.86 | 3.37 | 42.83 | 3.89 | 44.84 | 2.56 | **0.194** |
|  | HG2 |  | 21.11 | 3.18 | 20.35 | 3.20 | 20.73 | 2.24 | 0.330 |
|  | HA2 |  | 22.36 | 4.10 | 24.98 | 4.11 | 23.67 | 2.88 | 0.322 |
|  | HU2 |  | 9.56 | 1.35 | 11.49 | 2.00 | 10.53 | 1.20 | **0.348** |
|  | RWX |  | 39.61 | 3.33 | 39.35 | 3.47 | 39.48 | 2.38 | **0.508** |
|  | RGX |  | 14.01 | 1.74 | 13.77 | 1.74 | 13.89 | 1.22 | 0.350 |
|  | RAX |  | 30.65 | 3.10 | 30.74 | 3.32 | 30.70 | 2.25 | **0.875** |
|  | RUX |  | 15.27 | 1.56 | 15.61 | 1.76 | 15.44 | 1.16 | 0.948 |
|  | RW5 |  | 44.56 | 3.16 | 45.57 | 3.64 | 45.07 | 2.39 | **0.545** |
|  | RG5 |  | 14.16 | 2.11 | 13.77 | 2.15 | 13.96 | 1.49 | **0.570** |
|  | RA5 |  | 28.27 | 3.24 | 28.25 | 3.42 | 28.26 | 2.34 | **0.667** |
|  | RU5 |  | 12.76 | 1.41 | 12.17 | 1.69 | 12.46 | 1.09 | **0.215** |
|  | RW2 |  | 52.39 | 3.64 | 52.36 | 4.07 | 52.38 | 2.71 | **0.934** |
|  | RG2 |  | 15.31 | 2.86 | 13.04 | 2.80 | 14.18 | 1.99 | *0.073* |
|  | RA2 |  | 23.96 | 3.63 | 23.35 | 3.64 | 23.66 | 2.55 | **0.775** |
|  | RU2 |  | 7.94 | 1.14 | 10.93 | 2.05 | 9.43 | 1.18 | 0.116 |
| Soil variables | pH | pH in KCl (0-14) | 6.50 | 0.27 | 6.84 | 0.25 | 6.67 | 0.18 | 0.130 |
|  | CON | conductivity (dS m^-1^) | 0.03 | 0.00 | 0.03 | 0.00 | 0.03 | 0.00 | **0.124** |
|  | C | total carbon (%) | 6.99 | 1.29 | 8.48 | 1.39 | 7.74 | 0.95 | 0.368 |
|  | SAN | sand (%) | 46.27 | 1.91 | 53.25 | 1.84 | 49.76 | 1.39 | ***0.006*** |
|  | SIL | silt (%) | 26.30 | 2.03 | 24.61 | 1.97 | 25.46 | 1.41 | **0.556** |
|  | CLA | clay (%) | 27.36 | 2.22 | 22.14 | 2.06 | 24.75 | 1.54 | **0.471** |
|  | SOC | organic C (%) | 1.42 | 0.08 | 1.37 | 0.05 | 1.40 | 0.04 | **0.841** |
|  | SOM | organic matter (%) | 2.44 | 0.13 | 2.36 | 0.08 | 2.40 | 0.08 | **0.841** |
|  | N | total nitrogen (%) | 0.16 | 0.01 | 0.14 | 0.00 | 0.15 | 0.00 | *0.049* |
|  | C/N | C/N ratio | 9.11 | 0.18 | 10.10 | 0.16 | 9.60 | 0.14 | *0.001* |
|  | HUC | humic C (%) | 0.42 | 0.02 | 0.42 | 0.03 | 0.42 | 0.02 | **0.778** |
|  | HOC | humic C / organic C | 32.16 | 2.20 | 31.78 | 2.25 | 31.97 | 1.56 | **0.919** |
|  | P_ex_ | available P (ppm) | 1.50 | 0.09 | 2.68 | 0.09 | 2.09 | 0.10 | *<0.001* |
|  | PHE | phenols (nmol cat mg) | 1.99 | 0.06 | 1.46 | 0.03 | 1.73 | 0.05 | *<0.001* |

Supplementary Table S5.

RLQ analysis decomposes the co-correlations between environmental variables and trait attributes, constrained by abundance. This dataset (see the Supporting Document 41598_2018_26493_MOESM2_ESM.xlsx) reports the original data used in the analysis. Data sources used for obtaining the trait values are reported in the following list.

Cerabolini, B.E.L., Brusa, G., Ceriani, R.M., De Andreis, R., Luzzaro, A. & Pierce, S. Can CSR classification be generally applied outside Britain? *Plant Ecol.* **210**, 253-261 (2010).

Cornelissen, J.H.C., Castro Diz, P. & Hunt, R. Seedling growth, al location and leaf attributes in a wide range of woody plant species and types. *J. Ecol.* **84**, 755-765 (1996).

Cornwell, W.K. et al. Global woodiness database. Data from: Three keys to the radiation of angiosperms into freezing environments. *Nature*. Dryad Digital Repository. http://dx.doi.org/10.5061/dryad.63q27/2 (2013).

Díaz, S. et al. The plant traits that drive ecosystems: Evidence from three continents. *J. Veg. Sci.* **15**, 295-304 (2004).

Green, W. USDA PLANTS Compilation, version 1, 09-02-02. (http://bricol.net/downloads/data/PLANTSdatabase/) *NRCS: The PLANTS Database* (http://plants.usda.gov, 1 Feb 2009) (2009).

Kleyer, M. et al. The LEDA Traitbase: a database of life-history traits of the Northwest European flora. *J. Ecol.* **96**, 1266-1274 (2008).

Klotz, S., Kühn, I. & Durka, W. BIOLFLOR – Eine Datenbank zu biologisch-ökologischen Merkmalen der Gefäßpflanzen in Deutschland. *Schr.reihe Veg.kd.* **38**, 1-333 (2002).

Kühn, I., Durka, W. & Klotz, S. BiolFlor – a new plant-trait database as a tool for plant invasion ecology. *Divers. Distrib.* **10**, 363–365 (2004).

Moles, A.T., Falster, D.S., Leishman, M.R. & Westoby, M. Small-seeded species produce more seeds per square metre of canopy per year, but not per individual per lifetime. *J. Ecol.* **92**, 384–396 (2004).

Paula S., M. et al. Fire-related traits for plant species of the Mediterranean Basin. *Ecology* **90**, 1420–1420 (2009).

Poorter, H., Niinemets, U., Poorter, L., Wright, I.J. & Villar, R. Causes and consequences of variation in leaf mass per area (LMA): a meta-analysis. *New Phytol.* **182**, 565-588 (2009).

Pignatti, S. *Flora d'Italia* (Edagricole, 1982).

Royal Botanical Gardens KEW. Seed information database (SID), Version 7.1. http://data.kew.org/sid/ (2008).

Wirth, C. & Lichstein, J.W. The imprint of species turnover on old-growth forest carbon balances - insights from a trait-based model of forest dynamics in *Old-growth forests: function, fate and value* (eds. Wirth, C., Gleixner, G. & Heimann, M.) 81-113 (Springer, 2009).

Zanne, A.E. et al. Three keys to the radiation of angiosperms into freezing environments. *Nature* **506**, 89–92 (2013).
